# 3D Imaging of the Comparative Anatomical Structure of the Ossa Cranii in Some Ruminants

**DOI:** 10.3390/vetsci13090897

**Published:** 2026-08-31

**Authors:** Saime Betül Baygeldi, Yeşim Aslan Kanmaz, Hamza Yurttaş

**Affiliations:** Department of Anatomy, Faculty of Veterinary Medicine, Fırat University, Elazığ 23119, Türkiye; yesim.aslan@firat.edu.tr (Y.A.K.); hamzayurttas092@gmail.com (H.Y.)

**Keywords:** ossa cranii, ruminants, comparative anatomy, 3D anatomy

## Abstract

Three-dimensional comparative descriptions of the ossa cranii across representatives of the *Camelidae*, *Giraffidae*, and *Bovidae* remain limited despite their relevance to comparative veterinary anatomy and clinical practice. Given the distinct structural variations and functional adaptations of these families, a descriptive comparison of ossa cranii morphology is needed to advance anatomical knowledge and establish species-specific reference data. This study compared the principal ossa cranii of camel, giraffe, cattle, sheep, and goat to identify conserved anatomical features and species-specific adaptations. High-resolution three-dimensional digital skull models were generated with a Creality Raptor blue laser scanner and processed using 3D Slicer (Version 5.6.2). Due to marked interspecific differences in skull size and cranial capacity, quantitative morphometric analyses were not feasible; therefore, comparisons were based on qualitative gross anatomical evaluation of the os occipitale, os sphenoidale, os parietale, os temporale, os frontale, os pterygoideum, os ethmoidale, and os vomer. Although the overall organization of the ossa cranii was conserved, distinct species-specific adaptations were identified. Camels exhibited a prominent crista sagittalis externa, a broad nuchal surface, an elongated os vomer, and the absence of the tuber faciale and processus cornualis. Giraffes showed well-developed ossicones, a pronounced frontoparietal prominence, enlarged frontal sinuses, modified occipital condyles, and elongated ethmoid and vomer bones. Bovids were distinguished by horn-related structures, frontal sinus configuration, the processus jugularis, bulla tympanica, and foramen supraorbitale. Ossa cranii morphology reflects both anatomic relationships and functional adaptations associated with feeding behavior, head posture, and muscle attachment. These findings provide comparative anatomical reference data that support veterinary anatomy education, diagnostic imaging, species-specific surgical planning, and future studies in functional morphology and paleontology.

## Simple Summary

This study examines the ossa cranii morphology of species in the families *Camelidae*, *Giraffidae*, and *Bovidae* (camels, giraffes, cattle, sheep, and goats) using modern 3D laser scanning and digital modeling. Given the extreme variation in skull size, a qualitative anatomical analysis of bone structure and morphology was conducted instead of morphometric measurements. The findings show that, despite preservation of the ossa cranii basic organization, each species has evolved distinct adaptations. Camels are characterized by a prominent crista sagittalis externa, the absence of horns, and an os vomer twice as long as in other species. Giraffes exhibit the most distinctive structure, with massive air sinuses that protect the brain from impact, unique ossicones that fuse to the skull later, and specialized occipital condyles that enable the head to be held upright. In large ruminants (cattle), an expanded frontal region and processus cornualis structures, which form the basis of the horns, are prominent. In small ruminants, however, the morphology of the sphenoid bone and the nasoincisive suture line specific to goats have been identified as distinguishing taxonomic criteria. These morphological variations reflect the functional needs of the species, such as feeding strategies, fighting behaviors, and environmental adaptations. The results of this study are relevant to veterinary anatomy education, the planning of surgical interventions, and radiology.

## 1. Introduction

The giraffe *(Giraffa camelopardalis*), known for its long neck and limbs, is the tallest living land mammal. These extraordinary morphological features are key adaptations that enable the species to feed on leaves from high tree branches. Consistent with its feeding behavior, the giraffe’s long, prehensile tongue, the absence of upper incisors, and the evolution of the lower canines into a two- or three-lobed structure suited for stripping leaves are among the striking specializations of its craniofacial structure [1,2].

The dromedary camel (*Camelus dromedarius*), on the other hand, is a ruminant species that primarily inhabits arid and semi-arid climate zones and has adapted to environmental stress to a high degree. It holds great economic and cultural significance due to its versatile uses, such as for milk, meat, cargo transport and riding. This ecological success is related not only to physiological mechanisms but also to anatomical features that enable adaptation to environmental conditions [3,4,5].

Cattle (*Bos taurus*) were domesticated approximately 10,000 years ago and have become among the most important farm animals worldwide. They have considerable economic value as a primary source of meat, milk, and other animal products [6,7]. In parallel, there is growing interest in the detailed anatomy of the head in veterinary anatomy, surgery, and clinical practice. In this context, the literature includes numerous studies examining the skull morphology of domestic species, including cattle, water buffalo, horses, sheep, and goats [8,9,10].

With rapid advances in technology, traditional two-dimensional (2D) imaging methods are increasingly being replaced by three-dimensional (3D) modeling techniques, which are more qualitative and precise. This digital transformation, which has brought about a paradigm shift in veterinary anatomy research, is widely used in many fields, such as plastic surgery, orthopedics, dentistry, and medical education [11,12,13].

While traditional gross anatomy and linear morphometry methods are subject to limitations such as human-perception-based error margins and the overlapping of complex structures, 3D laser scanning and CT-based reconstruction methods enable the non-invasive examination of anatomical structures with high accuracy and reproducibility.

This study aims to meet modern standards for anatomical analysis by using high-resolution digital models that can document morphological variations and characteristic bone formations between species down to the finest detail [11,12,13].

The skull is one of the most complex structures in the skeletal system, protecting the central nervous system and sensory organs while housing the initial structures of the digestive and respiratory systems. The skull’s shape, the organization of its bones, and its morphometric characteristics vary significantly across species, reflecting feeding habits, habitat, and functional adaptations. Therefore, a detailed examination of skull morphology provides valuable information not only for comparative anatomy but also for numerous disciplines, including clinical diagnosis, surgical procedures, paleontology, and biological anthropology.

In recent years, three-dimensional imaging techniques and high-resolution computed tomography have significantly advanced the non-invasive study of cranial anatomy. However, systematic, three-dimensional comparative studies of ossa cranii structures across species in the *Camelidae*, *Giraffidae*, and *Bovidae* families remain limited. Existing research has primarily focused on a single species, and detailed information on morphological variation across these families is still lacking. This knowledge gap constrains not only comparative anatomy studies but also veterinary radiology, surgical planning, and the development of species-specific anatomical references. The main objective of this study is to examine the higher-level taxonomic and morphological differences among the *Camelidae*, *Giraffidae*, and *Bovidae* families through 3D models. Given the general consensus in the literature that morphological variation between species is more pronounced than intraspecific sex-based differences (sexual dimorphism), it is anticipated that this will have a limited impact on the study’s core taxonomic conclusions. However, the archival nature of the materials, the inability to access sex information for the specimens, and the extremely limited sample size, particularly in species exhibiting sexual dimorphism such as giraffes, are considered methodological limitations that hinder in-depth analysis of individual variations. Therefore, while it is not possible to make definitive and generalizing judgments about the entire population, it is believed that the descriptive findings obtained can contribute to the documentation of the relatively stable, coarse anatomical organization at the family level and provide a qualitative reference to the modern veterinary anatomy literature. The primary objective of this study is to identify species-specific morphological differences in the ossa cranii of camels, giraffes, cattle, sheep, and goats, which exhibit distinct feeding strategies, defense mechanisms, and lifestyles, using 3D models, and to provide the modern veterinary anatomy literature with a descriptive visual reference dataset.

## 2. Materials and Methods

### 2.1. Ethical Approval and Material Acquisition

This study was conducted in accordance with ethical approval dated 16 April 2025, and numbered 2025/06-02 (33612), obtained from the Local Ethics Committee for Animal Experiments at Fırat University.

The materials used in this study were skulls from adult, healthy individuals preserved in the Bone Archive of the Department of Anatomy at the Faculty of Veterinary Medicine, Fırat University. Specimen labels from the archived collections did not include sex data, making it impossible to definitively determine the sex of the entire sample group.

The sample group consisted of 1 giraffe (*Giraffa camelopardalis*), 2 dromedaries (*Camelus dromedarius*), 5 sheep (*Ovis aries*), 5 goats (*Capra hircus*), and 5 cattle (*Bos taurus*) skulls, selected without regard to sex.

“The main objective of this study is to highlight higher-level taxonomic and morphological differences among the *Camelidae*, *Giraffidae*, and *Bovidae* families using 3D models. The literature generally accepts that morphological variation between species (e.g., comparisons between giraffes and sheep) is much more pronounced than intraspecific sex-based differences (sexual dimorphism), and it is predicted that this will not alter the study’s main taxonomic findings.”

However, the inability to fully access sex information for the samples, due to the archival source of the materials, and the very limited number of samples, especially in species exhibiting significant sexual dimorphism such as giraffes, should be considered a methodological limitation that restricts the research’s capacity to analyze individual variation. Although the small number of giraffe samples (*n* = 1) makes it difficult to draw definitive conclusions about the overall population of this species, the qualitative findings remain important for documenting the stable anatomical organization of the formations in the sample examined and for providing a comparative reference to the modern veterinary anatomy literature.

The extreme differences in cranial capacity and absolute physical dimensions among the studied families (*Camelidae*, *Giraffidae*, and *Bovidae*) made direct digital (morphometric) processing of these structures unsuitable. Furthermore, due to structural differences in this distant taxonomic region, determining the homologous points (turning points) necessary for geometric morphometric analysis required adopting purely qualitative comparative principles of coarse anatomy instead of morphometric measurements.

### 2.2. Macroscopic Evaluation and Photodocumentation

The skulls were examined using gross anatomical methods to identify morphological variations and characteristic bone formations. High-resolution photographs were taken with a Nikon D3100 digital camera (Nikon Corporation, Tokyo, Japan) and an 18–55 mm lens to document the appearance of the bone structures in standard anatomical planes (dorsal, ventral, lateral, and caudal). Lighting was optimized during the photography sessions to prevent shadows and enhance detail.

### 2.3. Three-Dimensional (3D) Laser Scanning and Digitization

A Creality Raptor multi-mode laser scanner (Shenzhen Creality 3D Technology Co., Ltd., Shenzhen, China) was used to produce high-precision digital models of the skulls. The scanning protocol was carried out with the following technical parameters:

- Accuracy: The blue laser mode (±0.02 mm at 100 mm) was selected for maximum accuracy.
- Scan Mode and Speed: HD (High Definition) and manual scan modes were used to ensure error-free capture of all anatomical details, including foramina and fine bony protrusions. The data rate was set to 20 frames per second (FPS).
- Resolution: To ensure that even microscopic morphological features could be recorded, the point spacing was set to a maximum of 0.2 mm.

### 2.4. 3D Modeling and Digital Data Processing

The raw point cloud data from the scanner were processed using EXScan Pro (v4.0.0.4) to generate digital mesh models. The resulting raw models were imported into 3D Slicer (v5.6.2) for surface smoothing, hole filling, and topological corrections. The final 3D models, optimized on this platform, were prepared for comparative analysis.

### 2.5. Comparative Anatomical Analysis and Terminology

Due to the extreme variation in cranial capacity and physical dimensions among the skulls, direct, meaningful comparisons of these structures are not possible. Accordingly, no morphometric measurements (linear measurements, indices, etc.) were taken, and numerical data were not used as a basis. The study is based entirely on descriptive gross anatomy principles, and morphological differences and similarities between species were evaluated qualitatively. Nomina Anatomica Veterinaria (NAV) [14] was used as the primary reference for naming anatomical structures.

## 3. Results

The ossa cranii consist of the occipital bone, sphenoid bone, pterygoid bones, ethmoid bone, vomer, temporal bone, parietal bone, and frontal bone.

### 3.1. Findings of Camel’s Ossa Cranii

The camel’s skull was irregularly pentagonal in outline and divided into four surfaces: the frontal, basal, nuchal, and lateral surfaces.

**Figure 1:**
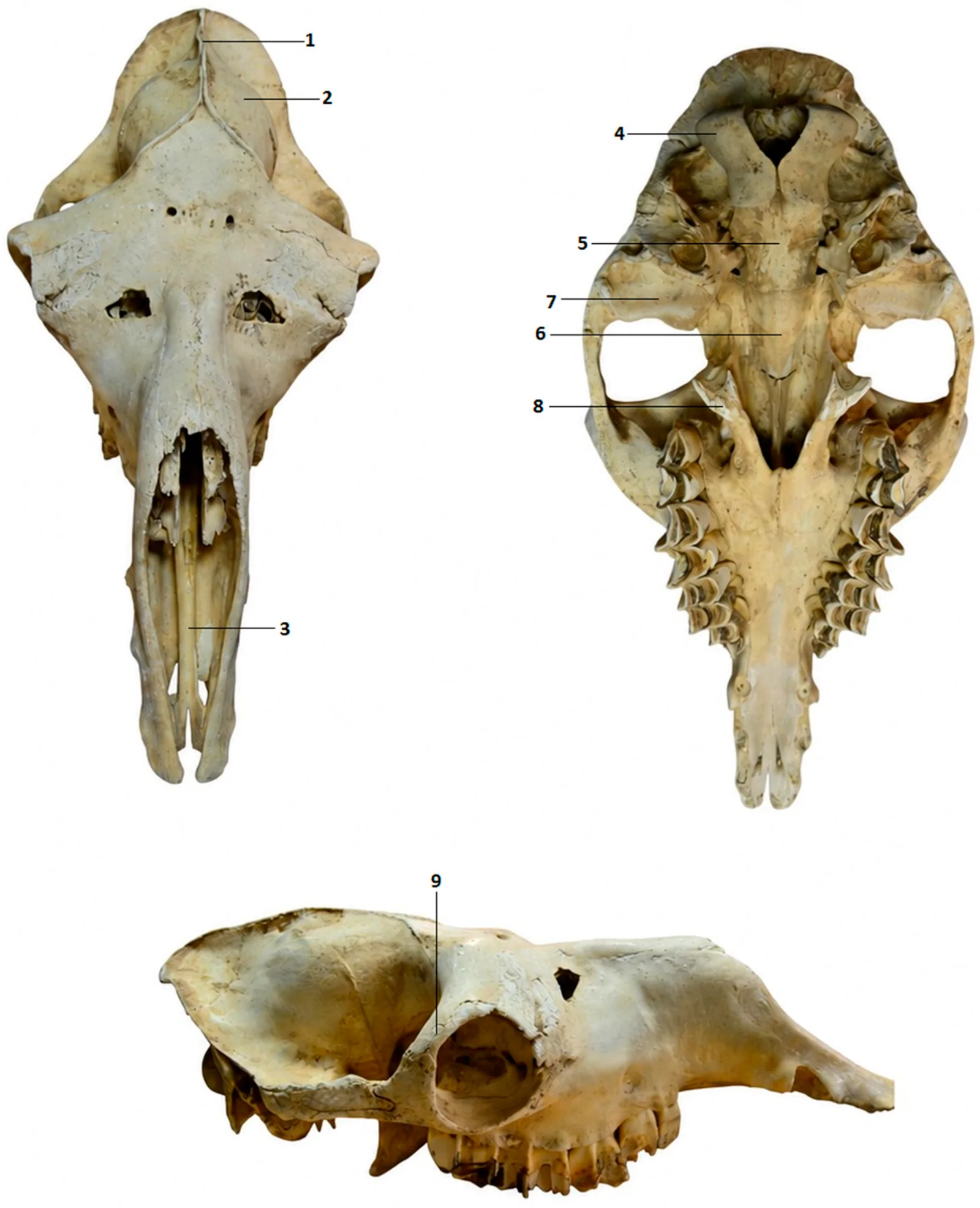
Images of a camel’s skull. 1. Crista occipitalis externa, 2. Os parietale, 3. Os vomer, 4. Condylus occipitalis, 5. Os occipitalis pars basilaris, 6. Os sphenoidale, 7. Tuberculum articulare, 8. Os pterygoideum, 9. Os frontale processus zygomaticus.

The occipital bone forms the entire nuchal (back of the neck) surface of the skull and extended toward the dorsal surface. The nuchal surface had a parabolic shape. The foramen mastoideum was quite large and was located within a deep fossa directly within the occipital bone. The condylus occipitalis was large, and the dorsal margin of the foramen magnum was notched at its center (Figure 1).

It was observed that the areas covered by the basisphenoid and presphenoid regions of the os sphenoidale were very close to each other.

The pterygoid bone is broad at the top and narrow and pointed at the bottom. It is a thin, plate-like bone located on each side of the choanae.

**Figure 2:**
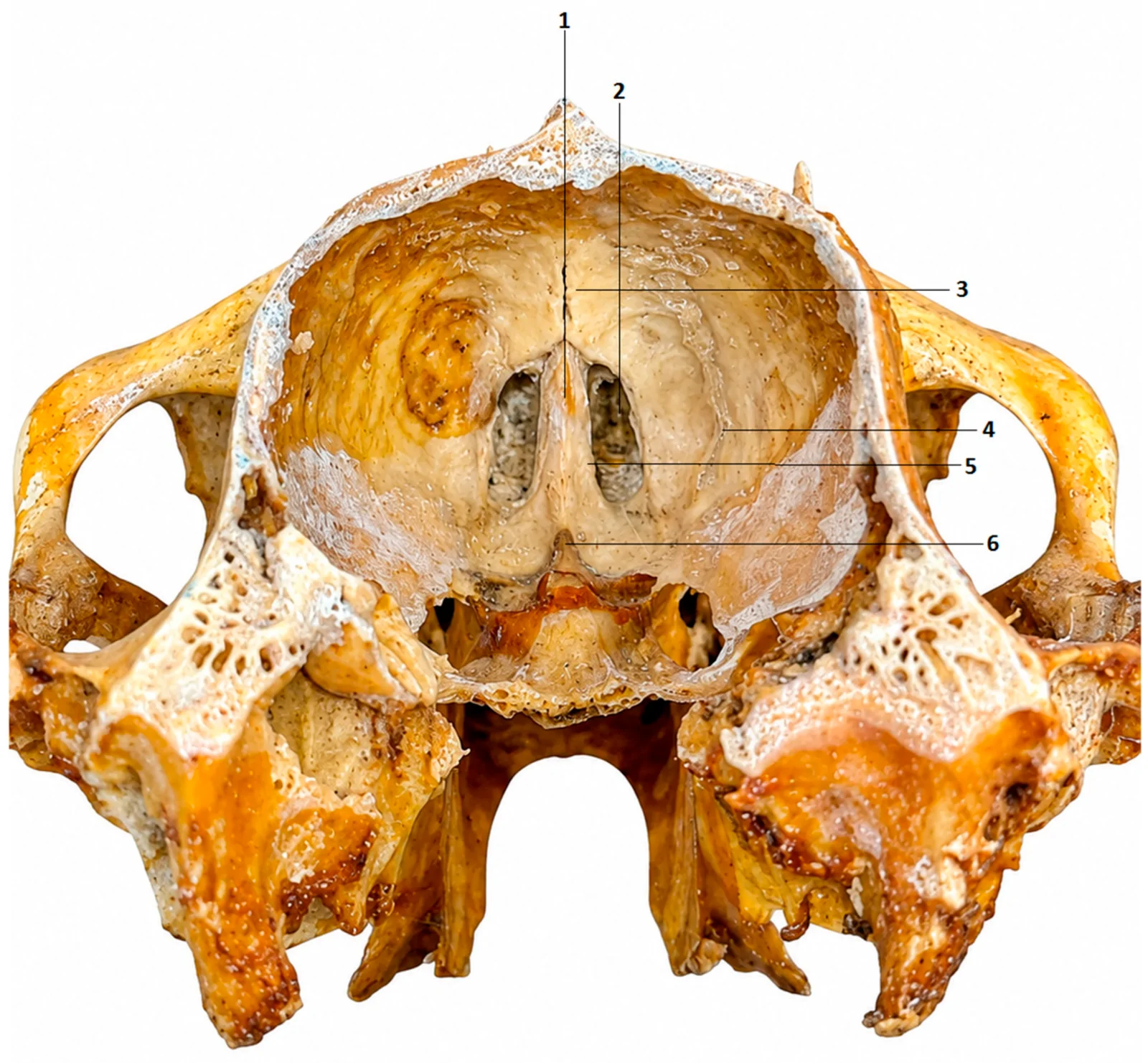
Images of a camel’s os ethmoidale. 1. Crista galli, 2. Lamina cribrosa 3. Lamina tectoria, 4. Lamina orbitalis, 5. Lamina perpendicularis, 6. Lamina basalis.

The ethmoid bone, as in the ox, forms the critical boundary between the cranial cavity (brain cavity) and the nasal cavity. Generally cone-shaped, this structure consists of a sieve-like, perforated plate (lamina cribrosa) that forms the anterior wall of the brain cavity and a small bony projection (crista galli) facing the cranial cavity (Figure 2). The vomer is long and narrow.

The bulla tympanica, located in the temporal bone, is flattened in the rostral–caudal direction. The processus mastoideus is fused with the base of the jugular (paracondylar) process. The tuberculum articulare was observed to be concave.

Structures such as the tuber faciale or the crista facialis were not observed in the camel.

**Figure 3:**
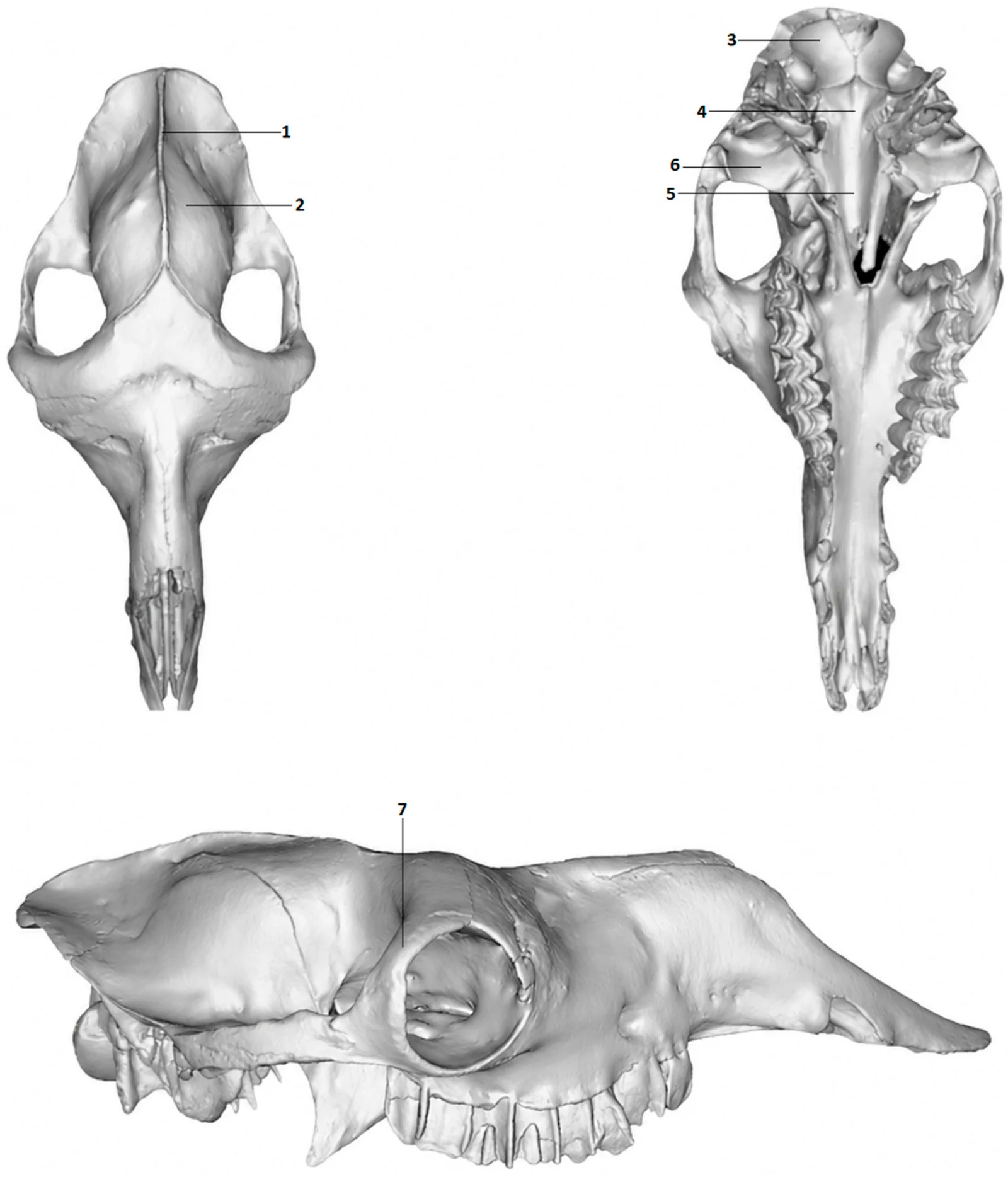
3D images of a camel’s skull. 1. Crista occipitalis externa, 2. Os parietale, 3. Condylus occipitalis, 4. Os occipitalis pars basilaris, 5. Os sphenoidale, 6. Tuberculum articulare, 7. Os frontale processus zygomaticus.

The most distinctive feature of the os parietale is the crista sagittalis (referred to as parietalis in some sources) externa, which forms a prominent, rough ridge approximately 1.5–2 cm high. The interparietal bone was found to resemble that of cattle (Figure 1 and Figure 3).

The os frontale is shorter than it is wide, and the orbit (eye socket) lies at the rostral–lateral margins of the bone. The processus cornualis was not observed in the camel. The foramen supraorbitalis appears as a deep fissure at the rostral–medial border of the orbit. The zygomatic process of the frontal bone alone forms the caudal wall of the orbit.

### 3.2. Findings of Giraffe’s Ossa Cranii

The crista occipitalis on the occipital bone is quite prominent. The condylus occipitalis was specialized in such a way that it was large and extended beyond the posterior edge of the occiput.

It was observed that the basisphenoid and presphenoid regions of the sphenoid bone were very close to one another. The presphenoid region of the sphenoid bone was divided into two parts: the corpus and the ala. However, the ala was determined to be very narrow.

The pterygoid bone is a small, slender, quadrilateral (four-sided) bone whose ventral end terminates in a Hamulus.

It cannot be said that the giraffe’s os ethmoidale has an anatomical structure completely different from that of other ruminants. In giraffes, the ossa cranii is set further back, while the splanchnocranium extends forward. As a result, the os ethmoidale forms a longer transition between the two sections.

In the temporal region, the tympanic membrane is small, irregular, and rough. The giraffe did not have a foramen lacerum.

The vomer in giraffes does not contain any unique anatomical structures. These differences are believed to primarily manifest as allometric (related to body and skull size) and functional adaptations. Due to the elongated facial skeleton, the vomer has become longer and thinner, thereby supporting the nasal septum over a greater distance.

**Figure 4:**
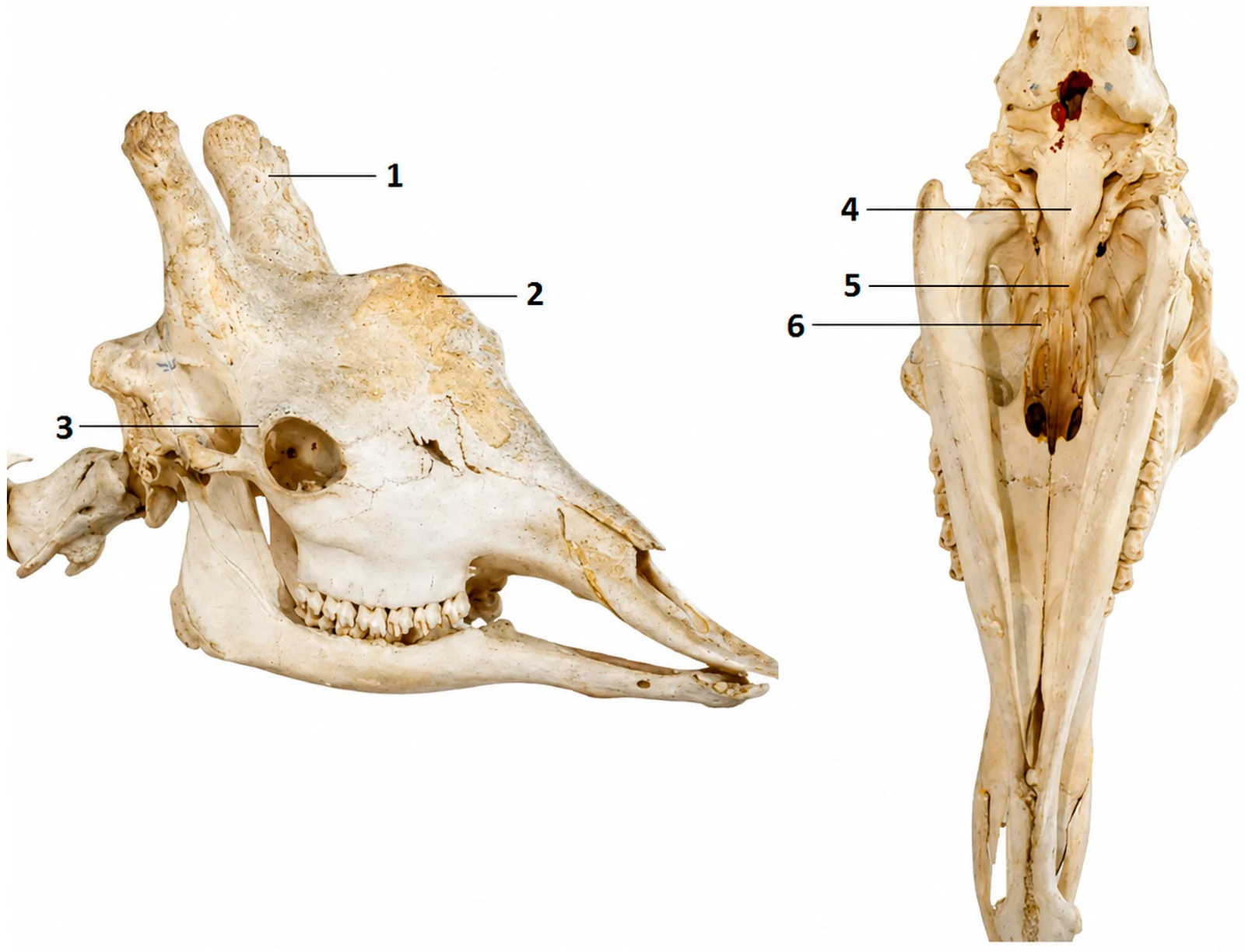
Images of a giraffe’s skull. 1. Ossicon, 2. Giraffacone, 3. Os frontale processus zygomaticus, 4. Os occipitale pars basilaris, 5. Os sphenoidale, 6. Os pterygoideum.

Giraffes have horn-like structures called ossicones (Figure 4).

The parietal bone is the origin of the giraffe’s characteristic pair of horns. This bone fuses with the frontal bone to form a broad, air-filled “frontoparietal boss.” The giraffe clearly lacks an interparietal bone.

The os frontale houses the frontal sinuses, the largest air cavities in the giraffe’s skull. The single, medium-sized forehead horn (giraffacone) sits on a protrusion composed primarily of the frontal bones and partly of the nasal bones. The orbital rims (eye sockets) are formed by this bone, and these rims contain sinus cavities. 

In the giraffe, the caudal wall of the orbit is formed by the zygomatic process of the frontal bone and the frontal process of the zygomatic bone (Figure 4).

### 3.3. Findings of Cattle’s Ossa Cranii

**Figure 5:**
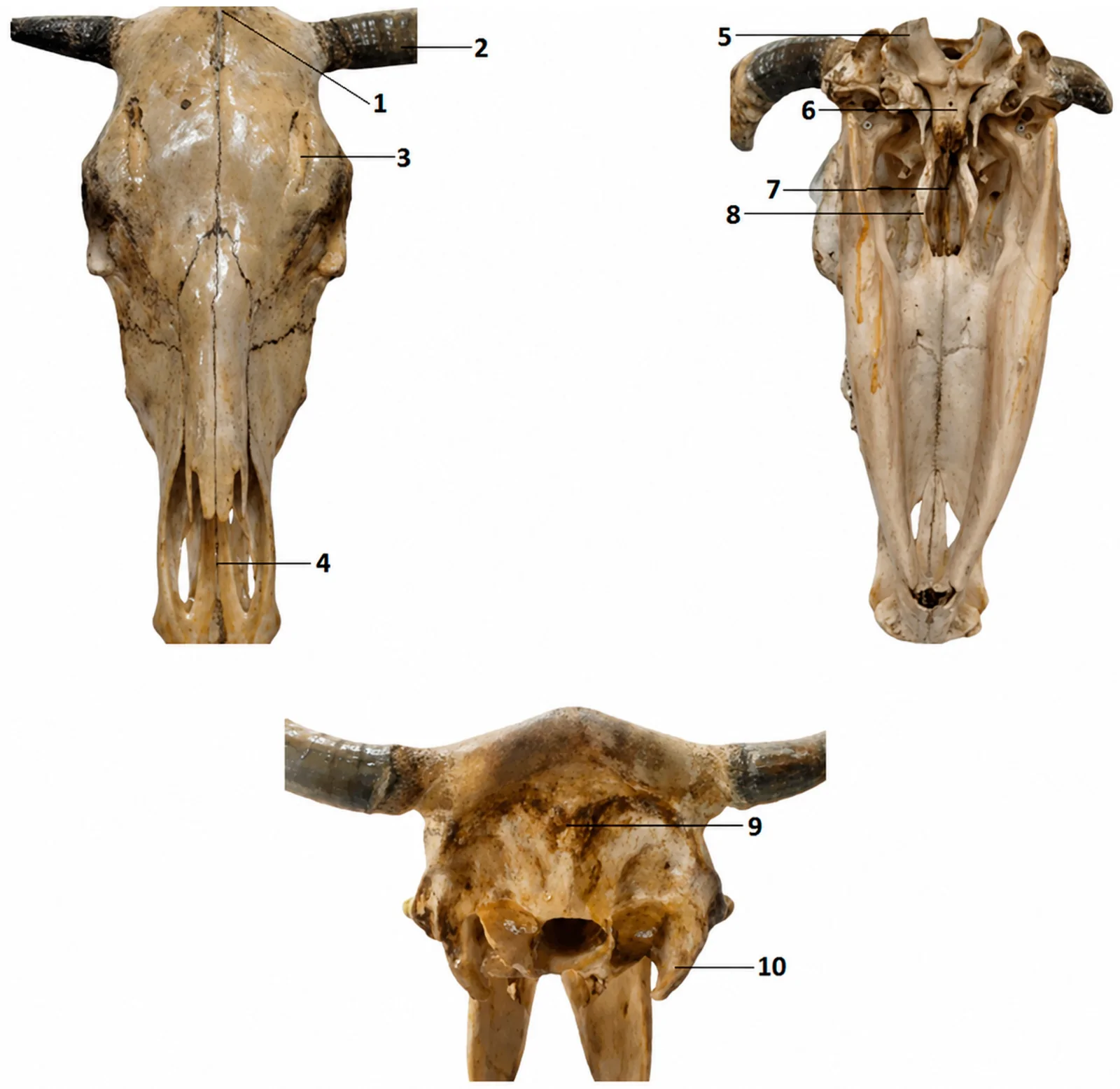
Images of a bovine skull. 1. Protuberentia intercornualis, 2. Processus cornualis, 3. Foramen (sulcus) supraorbitalis, 4. Os vomer, 5. Condylus occipitalis, 6. Os occipital pars basilaris, 7. Os sphenoidale, 8. Os pterygoideum, 9. Linea nuchae, 10. Processus jugularis.

The jugular process extends beyond the occipital condyles and is distinctly curved inward. The squama occipitalis is separated from the linea nuchae and continues, merging with the linea temporalis. The linea nuchae is located on the protuberantia occipitalis externa (Figure 5).

Os sphenoid consists of two main parts: the basisphenoid and the presphenoid. The presphenoid forms the rostral third of the skull base, while the basisphenoid contains the hypophysial fossa and the dorsum sellae.

Although the pterygoid bones are classified as cranial bones, they are located near the facial region. In cattle, they are a pair of flattened bones. 

**Figure 6:**
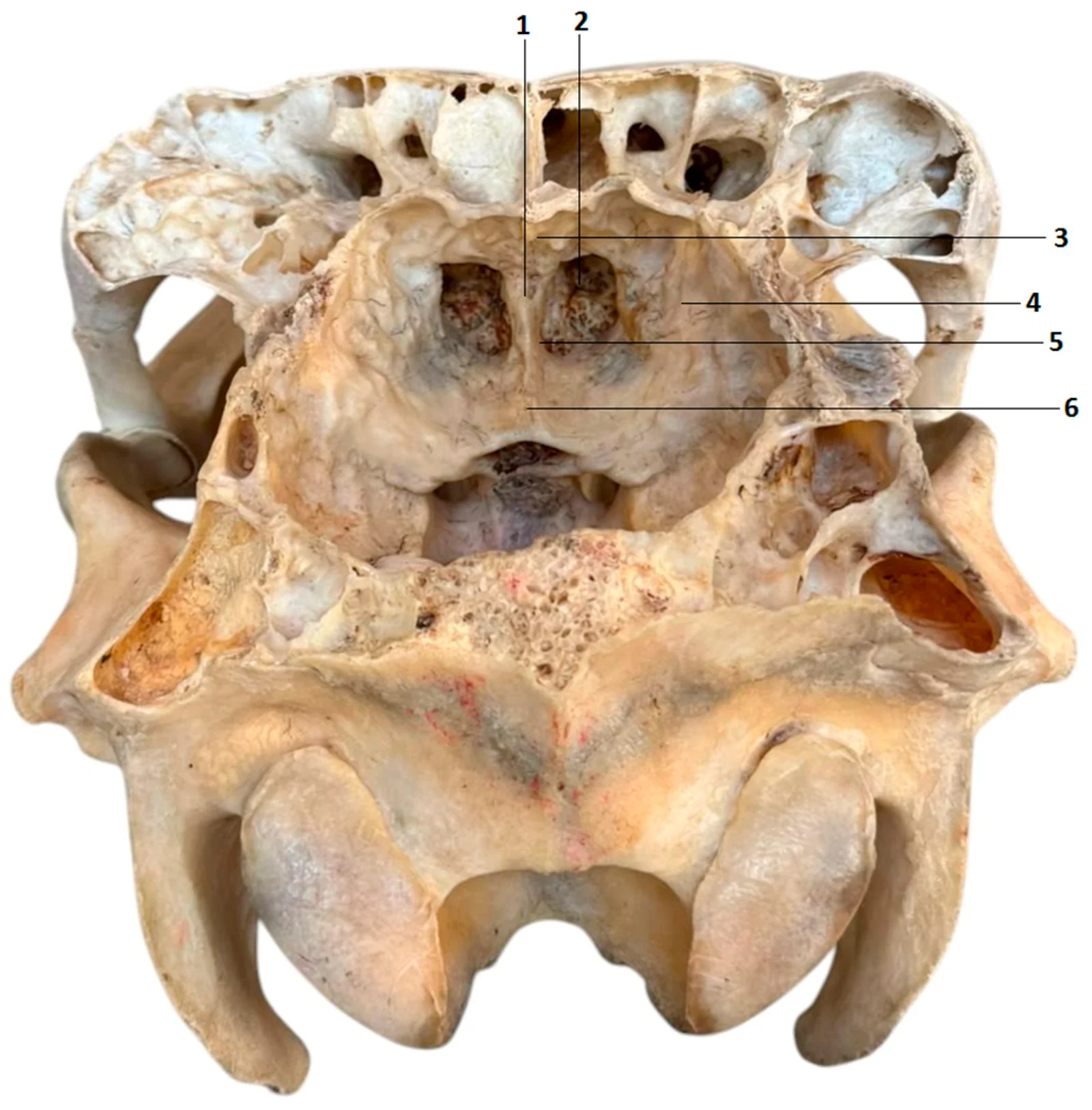
Images of a cattle’s os ethmoidale. 1. Crista galli, 2. Lamina cribrosa 3. Lamina tectoria, 4. Lamina orbitalis, 5. Lamina perpendicularis, 6. Lamina basalis.

Os ethmoidale. Although it is a single bone of the ossa cranii, it is largely located in the facial region (nasal cavity) (Figure 6).

Os vomer is the longest cranial bone in cattle and extends from the base of the skull to the apex. In cattle, the lower margin of the vomer does not fuse with the palatine portion of the palatine bone (os palatinum). The vomer canal is present.

The tympanic bulla, located medial to the styloid process of the temporal bone, appears as a downward-extending bulge. The external auditory meatus in cattle is tubular and extends laterally. The articular tubercle is flattened.

The parietal bones are a pair of bones that surround the cranial cavity. In cattle, they contribute to the formation of the nuchal surface (back of the neck).

**Figure 7:**
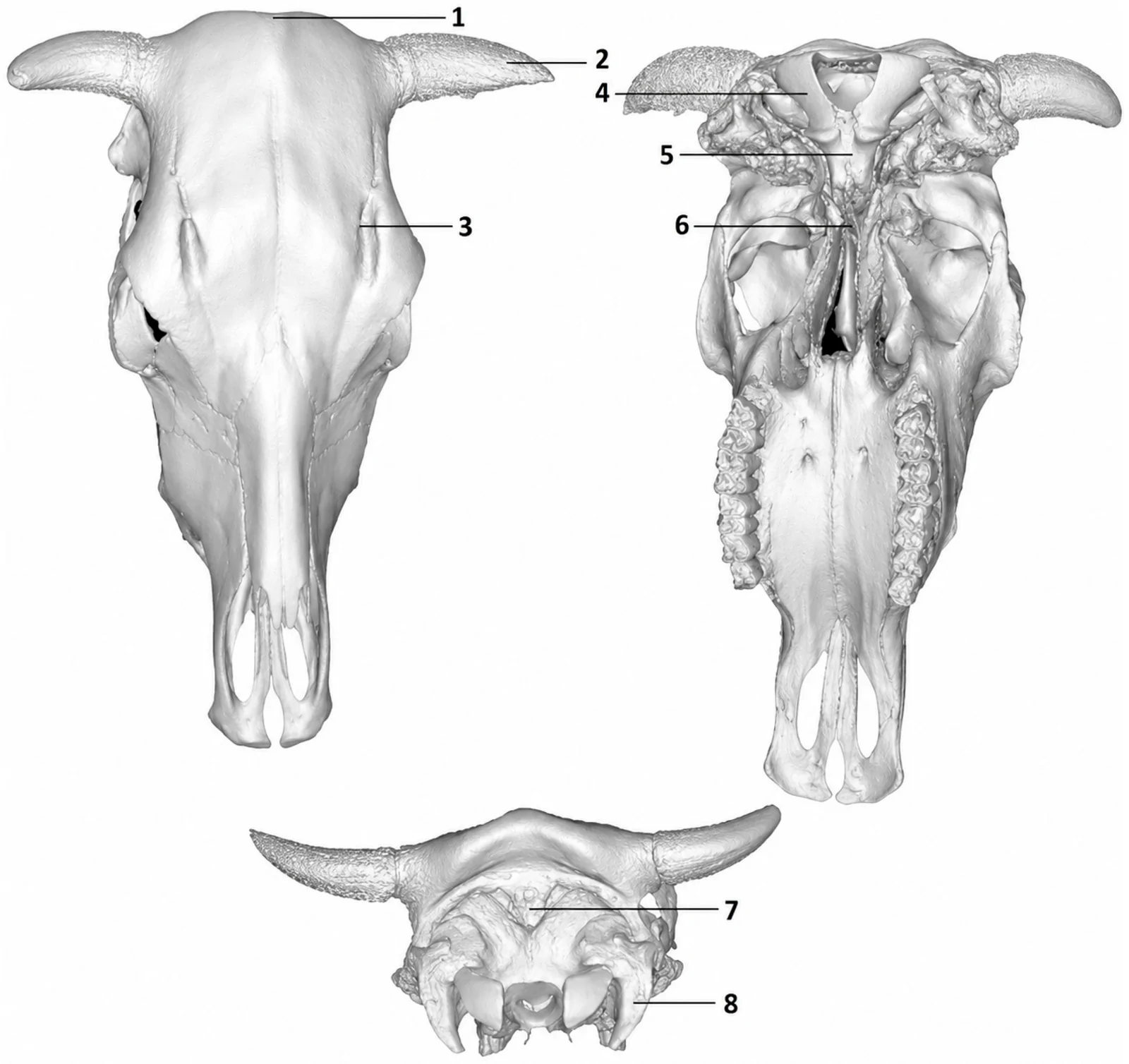
3D Images of a bovine skull. 1. Protuberentia intercornualis, 2. Processus cornualis, 3. Foramen supraorbitalis, 4. Condylus occipitalis, 5. Os occipital pars basilaris, 6. Os sphenoidale, 7. Linea nuchae, 8. Processus jugularis.

The frontal bone is the most prominent bone of the bovine skull; it forms a large portion of the skull vault (calvaria) and contains a large air cavity called the frontal sinus. In cattle, a protrusion (protuberantia intercornualis) lies between the horns. In the cattle, the caudal wall of the orbit is formed by the zygomatic process of the frontal bone and the frontal process of the zygomatic bone (Figure 5 and Figure 7).

### 3.4. Findings of Sheep’s and Goat’s Ossa Cranii

**Figure 8:**
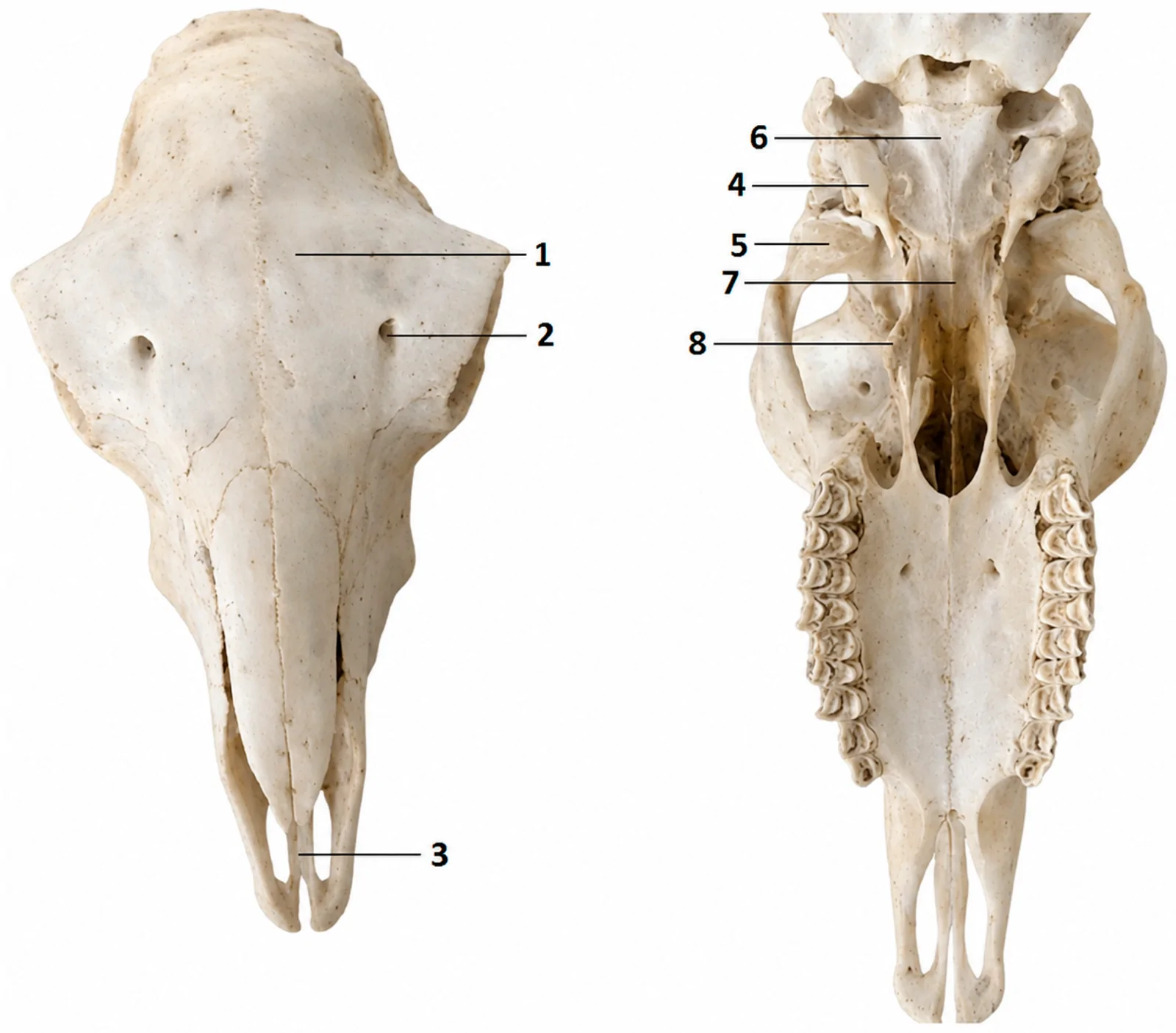
Image of a sheep’s skull. 1. Os frontale, 2. Foramen supraorbitale, 3. Os vomer, 4. Bulla tympanica 5. Tuberculum articulare, 6. Os occipitale pars basilaris, 7. Os sphenoidale, 8. Os pterygoideum.

The tuberculum musculare, located on the basilar part of the occipital bone, is prominent in sheep, whereas in goats this structure is less prominent. The processus jugularis is well-developed and directed caudolaterally (Figure 8).

The sphenoid bone in sheep is larger, longer, and wider than in goats. The width difference is primarily concentrated in the basisphenoid (os basisphenoidale) portion (Figure 8). The pituitary fossa (fossa hypophysialis) located on the basisphenoid bone has been identified in both species.

The hamulus portion of the pterygoid bone (hamulus pterygoideus) contributes to the formation of the lateral wall of the choana.

**Figure 9:**
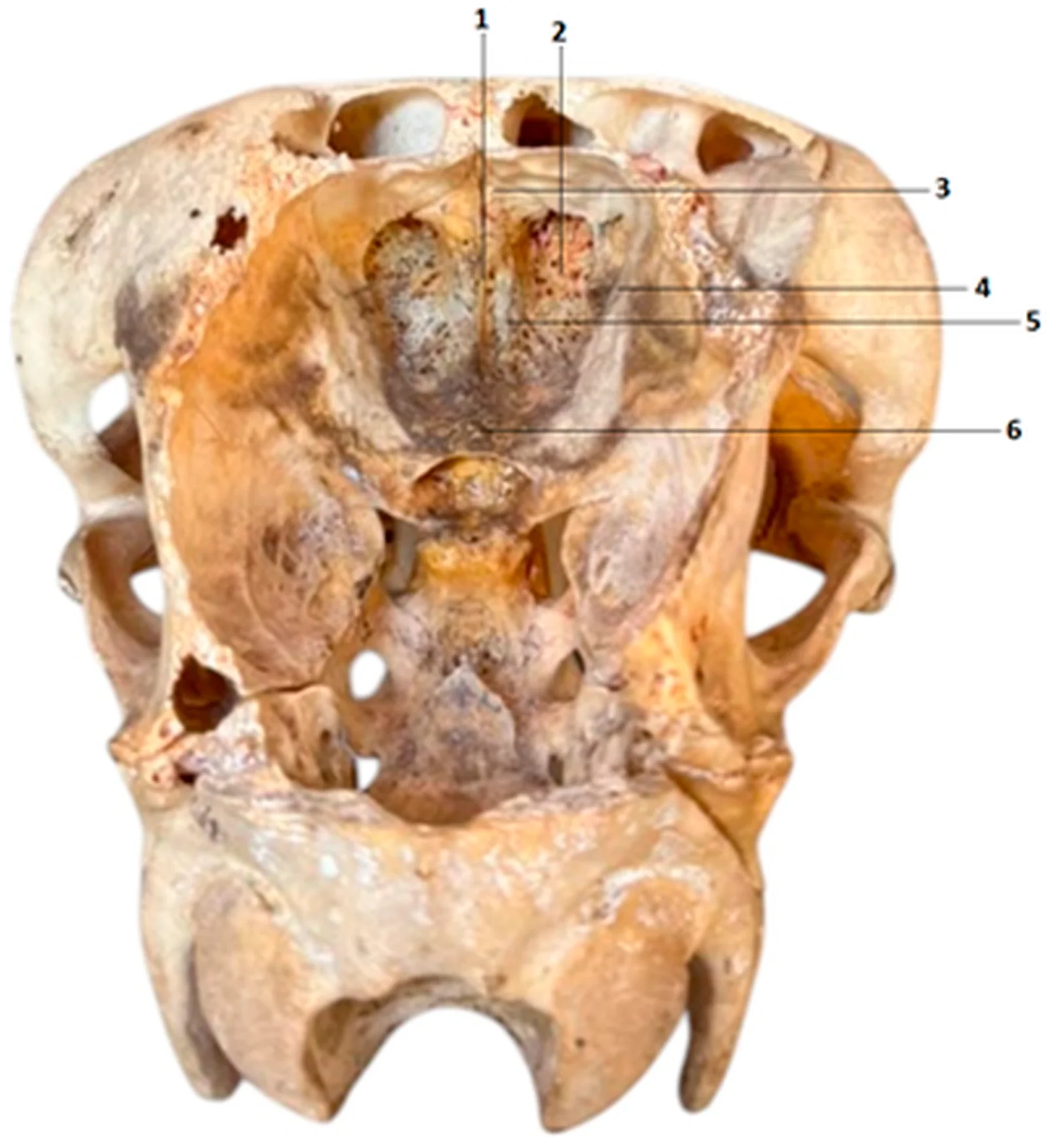
Images of a sheep’s os ethmoidale. 1. Crista galli, 2. Lamina cribrosa 3. Lamina tectoria, 4. Lamina orbitalis, 5. Lamina perpendicularis, 6. Lamina basalis.

The lamina cribrosa of the ethmoid bone is the primary structure that separates the nasal cavity from the cranial cavity (Figure 9).

Although the vomer is classified as a cranial bone, it lies entirely within the facial region.

**Figure 10:**
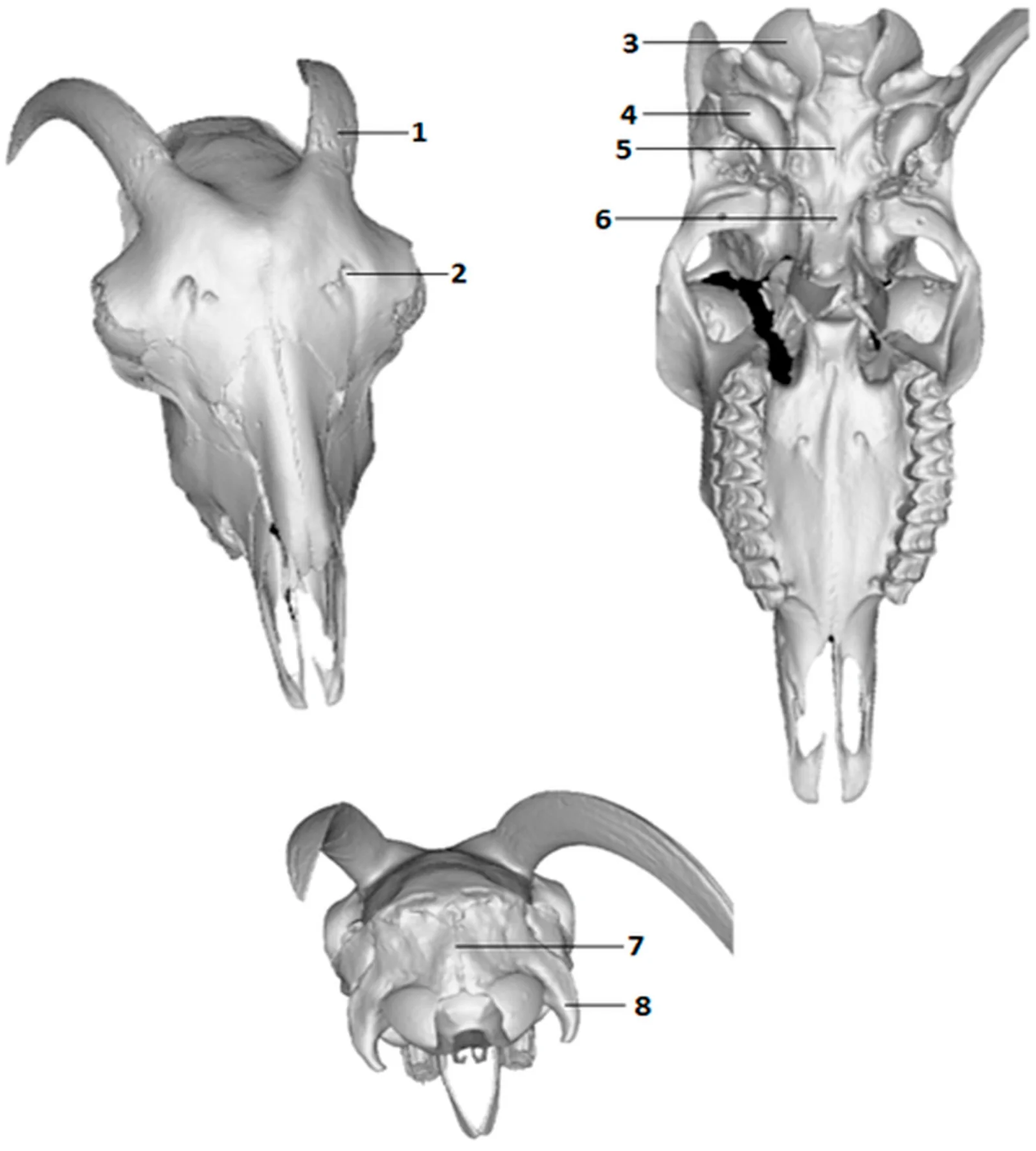
3D image of a goat’s skull. 1. Processus cornualis, 2. Foramen supraorbitale, 3. Condylus occipitale, 4. Bulla tumpanica, 5. Os occipitale pars basilaris, 6. Os sphenoidale, 7. Protuberentia occipitalis externa, 8. Processus jugularis.

In the temporal bone, the external auditory meatus (porus acusticus externus) is directed laterally in both sheep and goats. The styloid process, located in the petrous portion of the temporal bone, lies at the midpoint of the lateral side of the tympanic bulla in both species. The tympanic bulla is more expanded ventrally in goats (Figure 8 and Figure 10).

The planum parietale of the parietal bone is rectangular in both sheep and goats. The coronal suture (sutura coronalis), where the parietal bone meets the frontal bone, forms a straight line in both species.

The supraorbital foramen in the frontal bone is a single opening in both sheep and goats. In goats, this foramen opens into the orbit via a long, “L”-shaped canal. In both species, the caudal wall of the eye socket is formed by the zygomatic process of the frontal bone and the frontal process of the zygomatic bone (Figure 8 and Figure 10).

The tuber faciale on the upper jaw is a highly prominent and characteristic topographic landmark in both sheep and goats.

In goats, the processus cornualis has shifted slightly toward the rear of the orbit (Figure 8).

Some of the interspecies findings are presented in Table 1.

## 4. Discussion

The present study confirmed that, although the camel ossa cranii retains the general structural organization observed in ruminants, it possesses several distinctive morphological characteristics that clearly differentiate it from members of the Bovidae. In agreement with previous reports [15,16,17,18], the foramen mastoideum was located within a deep fossa of the occipital bone rather than at the junction of the temporal and occipital bones, as typically observed in cattle.

Similarly, the relatively elongated pars petrosa and the rostrocaudally flattened bulla tympanica distinguished the camel skull from that of bovids. Another characteristic finding was the absence of the processus cornualis, together with the presence of a deep, fissure-like foramen supraorbitale.

These features align with earlier anatomical descriptions and further support the view that Camelidae exhibit a distinct cranial morphology despite their superficial resemblance to ruminants. Such differences likely reflect the independent structural variations in camelids and may be linked to functional adaptations involving head biomechanics, sensory structures, and ecological specialization. The present findings therefore reinforce the value of comparative three-dimensional anatomical studies for distinguishing conserved cranial architecture from lineage-specific modifications among large herbivorous mammals.

The broad structure of the supraorbital fossa and the absence of vital vascular–nerve bundles in this region provide a safe access point for surgical procedures into the orbital cavity (eye socket) [19].

The present study demonstrated marked differences in the occipital, parietal, and temporal regions of the ossa cranii of camels and giraffes, reflecting both phylogenetic divergence and functional specialization. In camels, the enlarged sphenoid sinus surrounding the cranial cavity has been suggested in the previous physiological literature to contribute to brain thermoregulation under extreme desert conditions. Although our 3D anatomical models clearly document the expansive morphology of this sinus, we did not perform in vivo physiological testing; therefore, this adaptation is presented here strictly as a structural correlation consistent with previously proposed functional models [20]. Although the present study did not evaluate physiological function, the observed morphology is consistent with this proposed adaptive role. In giraffes, the enlarged condyli occipitales and a prominent pars basilaris ossis occipitalis provide a massive surface area that is morphologically consistent with the attachment of powerful cervical muscles and ligaments required to support an elongated neck. Because biomechanical performance (such as stress, strain, or muscle force) was not directly measured or modeled in this study, the functional relationship between these osteological features and neck biomechanics remains a plausible interpretation supported by the literature, rather than a directly tested finding [21,22]. The absence of the foramen lacerum in both camels and giraffes further distinguishes these species from horses and highlights interspecific variation among large herbivorous mammals.

The morphology of the parietal bone also varied considerably among the examined species. Consistent with previous studies, the giraffe had a single, broad, caudally positioned parietal bone, whereas paired parietal bones have been described in cattle, goats, horses, pigs, and dogs [23,24]. Likewise, the presence of ossicles at the frontoparietal junction in giraffes, contrasted with the complete absence of the processus cornualis in camels, further emphasized the distinct cranial morphology of these two species [25]. Differences in the bulla tympanica, which was rostrocaudally flattened in camels but smaller and irregular in giraffes [22], further support the existence of distinct lineage-specific ossa cranii adaptations. Collectively, these observations demonstrate that, despite a conserved basic ossa cranii organization, camelids and giraffids have evolved characteristic cranial modifications likely associated with their unique ecological and functional requirements.

The present observations further demonstrate marked interspecific variation in the morphology of the nasal and paranasal regions. In the giraffe, the ventral margin of the nasal septum articulates with the canal formed by the os vomer, a configuration that more closely resembles that reported in camels and buffaloes than in cattle [20]. In addition, the choanae differ considerably between camelids and giraffids, being plate-like in camels but smaller and associated with a distinct hamulus in giraffes.

These findings indicate that, despite sharing a common ruminant cranial plan, species-specific modifications of the caudal nasal cavity have evolved among large herbivorous mammals.

The giraffe exhibited the most distinctive ossa cranii morphology among the species examined. Its enlarged ossa cranii, extensive paranasal sinus system, and caudally positioned unpaired parietal bone are consistent with previous anatomical descriptions [20,26,27]. These features have been proposed as adaptations to the exceptional biomechanical demands of the elongated necks and the head-to-head combat behavior observed in adult males, although their functional significance warrants further biomechanical investigation.

In contrast, bovids were characterized by well-developed processus cornuales arising from the frontal bone and, particularly in cattle, by the protuberantia intercornualis, a topographical feature regarded as characteristic of this species [26]. The frontal sinus was also highly developed in cattle and has been reported to expand into adjacent cranial bones with advancing age. Although camels possess relatively large frontal and sphenoid sinuses, their degree of pneumatization was less extensive than in giraffes [27]. Collectively, these findings emphasize that variation in the ossa cranii reflects both anatomical divergence and functional specialization, providing a comparative framework for interpreting cranial adaptations in large herbivorous mammals.

The present study identified substantial variation in the morphology of the parietal, occipital, and vomer bones across the examined species, despite the overall conservation of ossa cranial organization. Consistent with previous anatomical descriptions, cattle had paired parietal bones that contributed to the roof and lateral walls of the ossa cranii, whereas the giraffe had a single, caudally positioned parietal bone resembling that reported in sheep rather than in other bovids [26,28]. This distinctive configuration is among the most characteristic ossa cranii features of the giraffe and may be associated with the unique biomechanical demands imposed by its elongated necks.

The present observations also confirmed marked differences in the occipital region. Previous studies have reported that the occipital region is broader in Simmental cattle than in Holsteins, providing a larger surface area for cervical muscle attachment [26]. Similarly, the modified occipital condyles observed in the giraffe have been suggested to permit a greater range of head movement relative to the cervical axis [28,29]. In contrast, camels were distinguished by a prominent crista parietalis externa (crista occipitalis externa) and by the position of the foramen mastoideum within a deep occipital fossa rather than at the temporo–occipital junction, consistent with previous reports [28,29,30,31].

Marked interspecific variation was also evident in the morphology of the os vomer. Although the vomer is the longest cranial bone in cattle, it is considerably more elongated in camels, reflecting the elongated nasal cavity characteristic of this species. In giraffes, fusion of the ventral margin of the nasal septum with the vomer canal produced a structural arrangement that more closely resembles that of camels than of cattle [27]. These findings indicate that the vomer exhibits lineage-specific morphological modifications despite its conserved role in supporting the nasal septum. Collectively, variation in the os vomer, together with differences in the occipital and parietal regions, demonstrates that the ossa cranii maintains a conserved structural framework while displaying lineage-specific morphological adaptations that likely reflect structural divergence and functional specialization.

This study, as a surgical approach, the tuber faciale (facial tubercle) defined as a surgical landmark is a critical topographic landmark that can be palpated even in live animals. This structure serves as a fundamental guide for determining the needle insertion point for an infraorbital nerve block (desensitization of the nostrils and upper lip), particularly in species such as cattle, camels, and buffalo [32,33]. The 3D anatomy of the temporal fossa and orbital region in camels is of vital importance for planning surgical access points during invasive procedures such as enucleation (removal of the eye) and tumor resection. Additionally, data on the location of the mandibular and mental foramina enable the accurate administration of mandibular nerve blocks during lower jaw fractures and tooth extractions [18]. Dehorning and frontal sinus trepanation procedures in cattle require a thorough understanding of the cornual process and the extent of the sinuses. It is thought that the 3D models obtained, showing the extension of the sinuses to the occipital region, can aid in surgical planning and contribute to the interpretation of diagnostic imaging findings [33].

Furthermore, regarding radiological imaging: Traditional radiography makes it difficult to visualize bone structures and clusters because of overlapping patterns. Our 3D models highlight complementary surface reference solutions for the existing radiological literature (CT/MRI studies) and complex ossa cranial structures in radiological images. These models are believed to aid surgical planning and contribute to the interpretation of diagnostic imaging findings [26].

Its contribution to paleontology and zooarchaeology lies in the difficulty of identifying species from fragmented skull pieces found at excavation sites. For example, the 3D morphology of the sphenoid bone provides a definitive criterion for distinguishing between sheep (*Ovis*) and goat (*Capra*) remains, which are morphologically very similar [29,30].

In anatomical education, the inner surfaces of the cranial cavity and complex foramen structures that are inaccessible through traditional cadaver-based training can be virtually examined using the 3D models we provide. This provides a non-invasive, repeatable, and highly accurate learning resource for medical and veterinary education [13].

The species examined in this study represent the families *Camelidae* (camels), *Giraffidae* (giraffes), and *Bovidae* (cattle, sheep, goats). Although all of these groups are ruminants, they do not form a monophyletic clade. It is widely accepted that the similar functional adaptations between members of the *Camelidae* clade (to which camels belong) and the *Ruminantia* clade (including giraffes and cattle) may have been inherited from a common ancestor approximately 55 million years ago, or, more likely, acquired independently (convergent evolution). This structural divergence is also reflected in skull morphology; the cranial structure of camels differs significantly from that of true ruminants. Therefore, rather than a monophyletic group analysis, this study aimed to compare species-based morphological variations in the ossa cranii structures of these members, which have different structural variations and feeding strategies.

## 5. Conclusions

The present study provides a descriptive, three-dimensional, comparative anatomical evaluation of the ossa cranii in representatives of the *Camelidae*, *Giraffidae*, and *Bovidae*. Although the overall ossa cranial organization was largely conserved among the examined species, distinct differences were identified in the morphology of the occipital, parietal, temporal, frontal, ethmoid, pterygoid, sphenoid, and vomer bones.

These variations demonstrate that, while a common mammalian cranial framework is maintained, lineage-specific modifications have evolved in response to distinct functional demands.

Among the examined taxa, camels and giraffes showed the greatest degree of ossa cranial specialization. The camel was characterized by features associated with facial skeleton elongation and robust cervical muscle attachment, whereas the giraffe displayed marked modifications of the frontoparietal region and occipital structures, consistent with the biomechanical demands of its elongated neck. In contrast, the ossa cranii of cattle, sheep, and goats retained the general bovid cranial pattern, with species-specific variation primarily associated with horn development and muscle attachment sites.

Rather than providing quantitative morphometric comparisons, this study establishes a qualitative, three-dimensional anatomical reference for the ossa cranii of these taxonomically distinct species. The findings contribute to comparative veterinary anatomy by documenting species- and group-specific cranial characteristics in camels, giraffes, cattle, sheep, and goats and by highlighting their potential structural and functional significance.

Comparative synthesis of the obtained 3D surface models shows that the studied species retain a common basic mammalian cranial plan but develop specializations that are stable at the family level. Camels (*Camelidae*) exhibit strong muscle attachment capabilities, characterized by a prominent crista sagittalis externa and a large nuchal surface, along with variable head and neck biomechanics. Giraffes (*Giraffidae*) display the most pronounced cranial specialization, with modified occipital condyles that aid in upright head attachment and enlarged frontal sinus systems that protect the brain from impacts. Members of the *Bovidae* family (cattle, sheep, goats) retain ruminant structures, including related sinus modules and processus cornualis structures. However, this analysis should be interpreted with caution, as it accounts for individual dimorphism and other gender-related differences, especially given the scarcity of specimens that are difficult to obtain, such as giraffes (*n* = 1) and camels (*n* = 2), compared to the members in the archives. Therefore, observed morphological features should not be generalized to broad evolutionary inferences and should be considered qualitative characterizations specific to the current settings.

In conclusion, this study indicates that the cranial morphology of taxonomic groups with significantly different cranial capacities and physical dimensions can be described using qualitative and three-dimensional methods, independently of the limitations of numerical morphometry. It is anticipated that the resulting digital models can serve as helpful reference sources in practical fields such as veterinary surgery, radiology, zooarchaeology, and anatomy education, and may provide a descriptive basis for future functional or biomechanical research.

## Figures and Tables

**Table 1:** A Comparison of Certain Ossa Cranii Structures. (+): available, (-): not available.

Animal Species	Crista Sagittalis Externa	Processus Cornualis	Caudal Wall of the Orbit	Os Interparietale	Foramen Supraorbitale	Distinguishing Morphological Features
Camel	(+) In the form of a distinct, rough ridge (1.5–2 cm high)	-	It is formed solely by the zygomatic process of the os frontale	+	In the form of a deep cleft at the rostral–medial border of the orbit	The facial tuber or facial crista is absent; the os vomer is quite long; it is located in a deep fossa within the foramen mastoideum occipitale bone.
Giraffe	-	(+) It is called ossicone.	It is formed by the zygomatic process of the frontal bone and the frontal process of the zygomatic bone	-	(+) There are spiral-shaped surface grooves radiating outward from this hole	It has large air sinuses; specialized occipital condyles; no foramen lacerum; and a single, large parietal bone structure.
Cattle	-	+	It is formed by the zygomatic process of the frontal bone and the frontal process of the zygomatic bone	+	+	The protuberantia intercornualis are located between the horns; there are large frontal sinuses; the os vomer is the longest cranial bone.
Sheep	-	+	It is formed by the zygomatic process of the frontal bone and the frontal process of the zygomatic bone	+	+	The muscular tubercle is prominent; the sphenoid bone is wider and longer than in goats; the facial tubercle is characteristic.
Goat	-	+	It is formed by the zygomatic process of the frontal bone and the frontal process of the zygomatic bone	+	+	The tympanic bulla is wider ventrally; the tuberculum musculare is less prominent; the nasoincisive suture line is taxonomic.

## Data Availability

The original contributions presented in this study are included in the article. Further inquiries can be directed to the corresponding author.
